# Influence of Plant Species on Microbial Activity and Denitrifier Population Development in Vegetated Denitrifying Wood-Chip Bioreactors

**DOI:** 10.3390/plants9030289

**Published:** 2020-02-26

**Authors:** Soheil Fatehi-Pouladi, Bruce C. Anderson, Brent Wootton, Sarah J. Wallace, Sonja Bissegger, Lloyd Rozema, Kela P. Weber

**Affiliations:** 1 Golder Associates Ltd., 1931 Robertson Road, Ottawa, ON K2H 5B7, Canada; 2 Department of Civil Engineering, Queen’s University, 58 University Ave., Kingston, ON K7L 3N6, Canada; 3 Centre for Advancement of Water and Wastewater Technologies, Fleming College, 200 Albert Street South, Lindsay, ON K9V 5E6, Canada; 4 Department of Chemistry and Chemical Engineering, Royal Military College of Canada, Station Forces, Kingston, ON K7K 7B4, Canada; 5 Aqua Treatment Technologies, 4250 Fly Road, Campden, ON L0R 1G0, Canada

**Keywords:** denitrification, wood-chip bioreactor, greenhouse, 16S rRNA amplicon sequencing

## Abstract

The microbial characteristics of four vegetated and one unplanted wood-chip bioreactors treating greenhouse effluent were investigated in a continuous experiment operated for over 2.5 years. The bioreactors were designed to reduce nitrate concentrations via naturally induced microbial denitrification. The vegetation type and reactor depth were both found to be significant factors in defining the mixed microbial activity. However, a consistent correlation between the abundance of the denitrifying communities and reactor depth could not be found across all reactors. The media samples from the unit planted with *Typha angustifolia* displayed higher microbial activities compared with the other reactors. This plant’s root-associated bacteria also demonstrated the greatest copies of the denitrifying genes *nirK* and *nosZ*. The most abundant denitrifier communities and those encoding the *nosZ* gene were found in the unplanted reactor, followed by the *T. angustifolia* unit. The *T. angustifolia* reactor demonstrated greater microbial activity and denitrification capacity at the depth of 20 cm, while the greatest denitrification capacity in the unplanted reactor was found at the depth of 60 cm. These findings indicated the importance of the *T. angustifolia* rhizosphere to support microbial community establishment and growth in the vicinity of the plant’s roots, although those populations may eventually develop in an unplanted environment.

## 1. Introduction

The successful application of denitrifying bioreactors to reduce nitrate levels in agricultural tile drainage has been previously documented and reviewed [1]. Denitrification refers to the reduction of nitrate by microbes to gaseous oxides of nitrogen and, ultimately, to dinitrogen gas. According to the common definition, the conversion of nitrate to nitrogen gas occurs via intermediates, including nitrite, nitric oxide and nitrous oxide. Denitrification is carried out by facultative heterotroph organisms which require a carbon source and can use either oxygen or nitrate as terminal electron acceptors. In a low-oxygen environment, denitrifying organisms use nitrate as their electron acceptor and, hence, nitrate can be reduced to nitrogen gas. In passive wood-chip denitrifying bioreactors, the carbon source required for successful denitrification is provided by the wood-chip media. 

The effective use of wood-chip denitrifying bioreactors for greenhouse effluent management and the positive influence of vegetation in enhancing nitrate reduction have been discussed in recent literature [2,3,4]. Denitrifying bioreactors were shown to remove between 19% and up to 88% nitrate in a long-term experiment [3], while the presence of *Typha angustifolia* resulted in the highest denitrification rate. The influence of microbial activities and potential functional capacity of denitrifying bacteria have been shown [4], where the *nirS* gene copies (as a functional biomarker for the populations with denitrifying capacities [5]), and the average well color development (AWCD) via community level physiological profiling (as an indicator of microbial activity) were positively correlated with the denitrification efficiency. In the previous study, however, the microbial assessment was conducted on the interstitial water samples. As a nondestructive method, the use of the interstitial water from inside the passive treatment systems is an advantageous and practical approach for spatial microbial studies [6]. As these systems are typically dynamic and mature over time, the more intrusive core sampling from the media inside the reactors to extract biofilm communities, which grow on the media, may compromise or alter the system’s internal microbial populations and their interactions. 

The activity of interstitial microbial communities is however often an order of magnitude less than those within biofilms [7]. The dissolved pollutants in the bulk water need to move to the vicinity of a solid surface, then diffuse through the ”stagnant water” layer and eventually penetrate the biofilm, where the pollutant removal takes place [8]. As such, the interstitial water samples from the immediate vicinity of the attached biofilm can likely give an idea of the major microbial communities present in the biofilm. However, factors such as water’s high mobility in flow-through units, potential short circuiting and mixing, as well as the preferential flow pathways, can all impact the characteristics of the interstitial water samples, making them less representative of the biofilm layer. Therefore, the characterization of the attached biofilm, instead of the interstitial water, where possible, can provide more realistic and representative information regarding the major communities responsible for denitrification.

Moreover, the vertical gradient (zoned layers) in passive vertical-flow systems such as constructed wetlands (CWs) can be influenced by a number of factors, such as the presence of plant roots, concentration of dissolved matter and other physiochemical parameters [9]. Hence, the microbial communities in different areas in a CW media might be different from one another. As described in Weber and Legge [7], for example, the rhizospheric microbial communities and the communities associated with the CW substrate are considered to be physiologically different from each other due to their different micro-environments. The authors further reported that the catabolic activity of the rhizospheric communities were much greater than the activity of the communities found in the gravel substrate. Thus, it is important to recognize and investigate the potential variation of the microbial communities and that of their activities in different zones of the vertical-flow denitrifying bioreactors. 

Since nitrite reductases (encoded by the *nirS* and *nirK* genes) and nitrous oxide reductase (encoded by the *nosZ* gene) are responsible for the multiple steps of the denitrification process, specific primers for these three genes have been used in assessing the denitrifying community composition in CWs [10]. These enzymes were used as *nirS* and *nirK* and are responsible for the reduction of nitrite to nitric oxide, while *nosZ* is responsible for the last step of the denitrification pathway by reducing nitrous oxide to nitrogen gas. For instance, the *nirS* functional genes were found in abundance in an effluent-fed CW [11], while the spatial variations of the *nosZ* gene were investigated in sediment samples from CWs [12,13]. Moreover, the growth of the bacteria containing the denitrifying gene *nirK* was reportedly enhanced in the subsurface CW microcosms containing *Typha latifolia* [14]. In a different CW planted with the same species, the potential nitrate reduction activities were shown to be significantly higher in the rhizosphere in comparison with the nonvegetated sediment, while the community structure of the denitrifying bacteria (*nosZ* gene) in the rhizosphere differed from that of the bulk sediment as well [15]. Therefore, it is expected that in the wood-chip bioreactors, whose biological denitrification mechanism is essentially similar to CWs, the microbial activities and denitrifying bacterial population would be different between the substrate (wood chips) and the root samples, and investigating these differences would provide crucial information for an improved and optimized design of denitrifying bioreactors.

In addition to the useful tools, such as community-level physiological profiling (CLPP) and quantitative polymerase chain reaction (qPCR), which are utilized to quantify the microbial activities and gene expression patterns, the study of the microbiome community structure in the water and biofilm samples using next-generation DNA sequencing is a powerful tool. 16S rRNA amplicon sequencing was used in this study to identify microbial taxa that could be contributing to nitrate reduction in our vegetated bioreactors. The microbial community of interstitial samples, described by Fatehi-Pouladi et al. [2], are also discussed in this study, so that the results from the solid (biofilm) and water phases can be readily compared.

For the previously noted reasons, it was of interest to evaluate the activity and potential function of microbial communities in our system. The main objective of this study was to investigate the developed attached (fixed) microbial communities in the hybrid denitrifying bioreactors that had been in continuous operation for over 31 months. The microbial assessment of the solid supporting media (wood chips) in the matured bioreactors (as the ideal environment for bacterial growth) is expected to provide useful information regarding the stratification of microorganisms and their spatial development through the reactors’ vertical profile.

In order to address this objective, this study aimed to determine the spatial microbial variations through the vertical profile of the vegetated bioreactors and the unplanted unit. Moreover, it was of interest to compare the microbial activities and population of functional genes in the reactors’ substrate (wood chips), as well as different plants’ rhizosphere to decipher the potential differences between the two environments and among different plant species. 

The second objective was to compare the activities of the solid-phase microbial communities with those of the interstitial water communities, discussed previously [2]. This comparison would allow examination of the two different sampling techniques and whether one can be replaced by another for reasonably comparable results.

The third important aim of this study was to analyze the microbial DNA of the water and biofilm samples in order to quantify the abundance of the major bacterial genera and species in both sample types. The key question was whether denitrification performance could be linked to the commonly known denitrifiers and to which major genera or species these microorganisms were affiliated. It was also of interest to compare these organisms between the bioreactors and analyze their composition shift in time and through reactor depth.

## 2. Materials and Methods

### 2.1. Bioreactors Set-up

The pilot-scale project was built and operated in the Department of Civil Engineering at Queen’s University (Kingston, ON, Canada). The experiment consisted of four vegetated and one unplanted wood-chip bioreactors. Each of the five bioreactors was constructed from open-top 220-L barrels (diameter: 56 cm, height: 90 cm) and filled with maple hard wood chips, acquired from Quebec, Canada, in a single 80-cm layer. 

The synthetic greenhouse effluent was prepared in the laboratory as per the common greenhouse effluent characteristics reported in the literature and by greenhouse operations in Ontario, Canada [4,16,17,18]. The average influent nitrate concentration was 307 mg N L^−1^ from day 1 to 387 and 202 mg N L^−1^ from Day 387 to 936. The effect of low and high nitrate loading has been discussed previously [2]. 

Each vegetated wood-chip reactor was individually planted with a different plant species, including softstem bulrush (*Schoenoplectus tabernaemontani* C.C. Gmel. Palla), narrowleaf cattail (*Typha angustifolia* L.), prairie cordgrass (*Spartina pectinata* Bosc ex Link) and saltgrass (*Distichlis spicata* L. Greene). The reactors operated in the saturated vertical-flow mode (top-bottom) and received synthetic greenhouse effluent at a constant flow rate of 30 L day^−1^. The total operation time of the experiment was 936 days. However, prior to day 188, switchgrass (*Panicum virgatum* L.) and Canada wildrye (*Elymus canadensis* L.) were in use in place of *S. pectinata* and *D. spicata*. The former species were replaced as they did not survive the experimental conditions. 

### 2.2. Sampling Methodology and Timeline

The interstitial water samples for DNA extraction, quantitative polymerase chain reaction (qPCR) and 16S rRNA amplicon sequencing were collected on seven occasions on days 147, 359, 389, 442, 517, 601 and 665. The interstitial water samples for mixed microbial community analysis were collected on day 531. The destructive core sampling was performed on day 936 (end of the experiment), when both wood-chip and plant root samples were collected. 

The interstitial water samples were collected using sterile individual dedicated syringes from the sampling tubes embedded in each reactor. A vertical PVC tube (inner diameter: 2.54 cm) had been positioned in the center of each reactor (planted and unplanted) through the wood-chip media as a platform to support 3 interstitial water sampling tubes (inner diameter: 1.6 mm), which were installed at 20, 40 and 60 cm below the top surface of the wood chips. 

The core and root sample collections at the end of the experiment were completed as follows. The reactors were first slowly drained from the outlet pipe using gravity and a low-flow peristaltic pump to minimize the disturbance to the attached biofilm. The plants were then removed, and 5 g of the below-ground biomass (roots) were collected using a sterile utility blade (2 replicates per sample). The average depth of the roots was estimated as approximately 30 cm below the media surface, although the length of the roots varied between the species. Twelve grams of intact wood chips were collected from the center of 3 depths through each reactor at 20, 40 and 60 cm below the media surface, avoiding any soil or other particles from the plant biomass (2 replicates per sample). The wood-chip layers were removed manually, while samples were picked by sterile tweezers. The root and wood-chip samples were transferred to sterile tubes and stored at 4 °C for less than 24 h before further processing. The sterilization of the tools between samples was performed using a 70% ethanol solution.

The drainable porosity (effective porosity) determined based on Equation (1) was determined as 0.57, similar to the porosity of 0.58 calculated earlier for fresh wood chips.

(1) $$n_{e}=\frac{V_{w}}{V_{r}}$$

where $n_{e}$ is the drainable porosity (dimensionless), $V_{w}$ is the volume (L) of the water drained from the bottom of a saturated bioreactor and $V_{r}$ is the volume (L) of the portion of the bioreactor filled with wood-chip media.

Additional wood-chip samples were placed in the oven at 70 °C for 42 h, and the moisture content was calculated as 63% based on the wet weight, according to Equation (2):

(2) $$MC=\frac{W_{water}}{W_{wood}}\times 100$$

where MC is the wet wood-chip moisture content (%), $W_{water}$ is the weight of the evaporated water (g, difference between the wood chips before and after drying) and $W_{wood}$ is the weight of the wet wood chips (g) before drying. The average wet weight of the wood chips in each barrel was measured as 97 kg, equaling to approximately 36 kg of dry mass.

The biofilm detachment was performed following the protocol adapted from [19]. The wood-chip and root samples were added to 250 mL Erlenmeyer flasks containing 150 mL of a phosphate buffer solution (PBS; 10 mM, pH of 7 with 8.5 g L^−1^ NaCl) using autoclaved deionized water. Samples were shaken in the PBS solution for 3 h at 150 rpm to suspend the attached biofilm in the liquid phase. The suspended solution was then divided in three parts for the next steps.

### 2.3. Mixed Microbial Community

First, 25 mL of each of the two replicate solutions were combined to produce a 50 mL sample volume for community-level physiological profiling (CLPP). Biolog® EcoPlates (BIOLOG Inc., Hayward, CA, USA) with 31 different carbon sources and one blank well in triplicates (96 wells per plate) were inoculated with each detached biofilm solution sample at room temperature on the same day the samples were shaken and approximately 24 h after sample collection. CLPP was performed in an aseptic environment using sterile laboratory consumables and a 70% ethanol spray for sterilizing the tools and bench tops. The unfiltered samples (100 µL) were pipetted to each well, after which, the plates were read for the first time (time = 0) and then stored in the dark during the incubation period. The optical densities of the plates were read using a microplate absorbance reader (TECAN Infinite®, Tecan US Inc., Morrisville, NC, USA) at an absorbance of 590 nm at several intervals (at 0 h, 21 h and then every 4 h until 129 h) following the inoculation. The CLPP data analysis was performed according to the previously established methods described by Weber et al. and Weber and Legge [20,21]. Richness was calculated as percentage values, described by Fatehi-Pouladi et al. [2]. The absorbance readings at 49 h were selected as a balance between the lowest numbers of the wells with an absorbance value of higher than 2 and the greatest standard deviations among the absorbance readings of the wells in each plate.

The absorbance values of each well in the EcoPlates were normalized prior to principle component analysis (PCA) and agglomerative hierarchical clustering (AHC). The normalized absorbance for each well was calculated according to [20] per Equation (3).

(3) $$\bar{A_{k}}=\frac{A_{k}-A_{0}}{\frac{1}{31}\sum_{i=1}^{31}\left(A_{i}-A_{0}\right)}$$

where $\bar{A_{k}}$ is the normalized absorbance reading of well k, $A_{k}$ is the absorbance reading of well k and $A_{0}$ and $A_{i}$ are the absorbance readings of the blank well and well i, respectively. In the event of a negative value for the blanked absorbance reading for a well $\left(A_{k}-A_{0}\right)$, the negative value was replaced by zero. After normalization, the data were transformed using the Taylor power law in order to improve the normality of the dataset. The Taylor power law stabilizes the variances and, thus, makes the data more likely to conform to the assumptions of parametric analysis, including normality [22]. The Taylor transformation assumes that the variances of the natural samples are proportional to a fractional power of the mean [23] Equation (4).

(4) $$S^{2}=a\times {\bar{m}}^{b}$$

where $S^{2}$ is the variance of a sample, $\bar{m}$ is the mean of a sample and a and b are characteristics of the population, which were determined by plotting $log(S^{2}$) versus $log(\bar{m}$).

### 2.4. Organic Content

For the total organic content analysis, 50 mL of the solution were separated. Samples were poured in porcelain crucibles and placed in the oven at 105 °C for 42 h until a constant dry weight could be reached. The dried samples were then ashed in a furnace oven at 550 °C for 20 min. The average total solids (TS) in the suspended solution was 9.5 g L^−1^ for wood chips and 11 g L^−1^ for roots. The total organic content (TVS, total volatile solids) of the detached biofilm was 0.4 g L^−1^ for wood chips and 0.7 g L^−1^ for roots. TS (g L^−1^) and TVS (g L^−1^) were determined according to Equations (5) and (6), respectively.

(5) $$TS=\frac{W_{105}}{0.050}$$

(6) $$TVS=\frac{W_{105}-W_{550}}{0.050}$$

where $W_{105}$ is the solid’s weight (g) after drying the biofilm solution in the 105 °C oven, 0.050 L is the sample volume and $W_{550}$ is the remaining solid’s weight (g) after burning the dried solution samples in the furnace oven at 550 °C.

### 2.5. DNA Extraction and qPCR

DNA extraction of the interstitial water samples was conducted according to [2]. As for the biofilm samples, between 25 to 75 mL of each suspended solution was filtered through nylon membrane filters (0.2 µm pore size, diameter: 47mm; Millipore, Etobicoke, ON, Canada), and filters with the captured residues were placed in sterile tubes and stored at −20 °C until further processing. The thawed filters were rolled inside the extraction vials of the FastDNA SPIN Kit for soil (MP Biomedicals, Santa Ana, CA, USA), and the DNA extraction was continued following the manufacturer’s protocol. The nucleic acid concentrations were measured using a NanoDrop 2000 spectrophotometer (Fisher Scientific, Toronto, ON, Canada).

The expression levels of the nitrite reductase genes (*nirK* and *nirS*) and nitrous reductase gene (*nosZ*) were determined by qPCR. The primers and programs used for the amplification of *nirK*, *nirS* and *nosZ* have been previously designed and optimized by Ligi et al. [24]. The samples were analyzed in 1:10 dilutions in duplicate on Eco™Real-Time PCR System (Illumina, San Diego, CA, USA) using Maxima SYBR Green Master Mix (Fisher Scientific, Toronto, ON, Canada) and 1.0 µM of forward and reverse primers. The reaction efficiencies calculated by EcoStudy Software (Illumina, San Diego, CA, USA) were 100% ± 10% with an R^2^ > 0.990. Standard curves were included on each qPCR plate of 6 serial dilutions (150, 30, 6, 1.2, 0.24 and 0.048 ng) of the pooled extracted DNA samples with an additional no template control (no DNA).

The gene quantities were calculated according to Equation (7), where X represents the sample’s normalized amplified target gene (*nirK* or *nirS* or *nosZ* or 16S rRNA) quantity (no unit), $X_{1}$ is the recorded amplified target gene quantity (ng) of the sample, 75 µL is the volume of the DES (DNase/pyrogen-free water) in the DNA extraction procedure and $N_{c}$ is the nucleic acid concentration (ng µL^−1^) of the respective sample after DNA extraction.

(7) $$X=\frac{X_{1}}{75\times N_{c}}$$

The number of gene copies normalized against the DNA content of each sample was calculated according to Equation (8).

(8) $$\mathrm{Number}\;\mathrm{of}\;\mathrm{copies}=\frac{X\times 6.0221\times 10^{23}}{N\times 660\times 10^{9}}$$

where X (no unit) is the amount of the normalized target gene for *nirK*, *nirS*, *nosZ* or 16S rRNA Equation (7); $6.0221\times 10^{23}$ molecules mole^−1^ is Avogadro’s number; N is the length of dsDNA amplicons (bp) derived from [24]; 660 g mole^−1^ is the average mass of 1 bp dsDNA and $10^{9}$ is the unit conversion of g to ng.

### 2.6. 16S rRNA Amplicon Sequencing

To assess the composition of the microbial community, the variable V3 and V4 regions of the 16S ribosomal RNA gene (16S rRNA) were amplified following the Illumina 16S Metagenomic Sequencing Library Preparation guide (version B, Illumina Canada Ulc., Victoria, BC, Canada). Refer to supplemental information (SI) document for further details on the sequencing preparation.

To sequence the samples using the MiSeq (Illumina Canada Ulc., Victoria, BC, Canada), a subset of 96 samples were each normalized to 0.5 nM and pooled before being denatured with 0.2N NaOH into single-stranded DNA. The denatured DNA was diluted and normalized to 3 pM with hybridization buffer (Illumina Canada Ulc., Victoria, BC, Canada). For quality control of the run, a 10% PhiX control library spike-in was similarly denatured, diluted and pooled with the DNA samples. The MiSeq Reagent Kit v3 for 600 cycles (Illumina Canada Ulc., Victoria, BC, Canada) was used for sequencing 2 × 300 bp paired-end reads. After a 60-h run time, 30.7 million total reads with 22.9 million reads passing filter were produced in a cluster density of 1182 k mm^−2^. Sixty-one point two percent of the forward read and 49.4% of the reverse read had a quality score of over Q30.

Raw fastq.gz files were input to Illumina’s BaseSpace and analyzed using the 16S Metagenomics app (Illumina, Inc.). This app uses the ClassifyReads algorithm (modified Ribosomal Database Project classifier) to match sequences to the Illumina-curated version of the Greengenes taxonomic database (May 2013). The abundances of each of the taxonomic levels, including the unclassified portions, were determined as percentage (%) of the total reads passing filter, therefore normalizing each sample to its own number of reads (N = min–max number of reads of 3189–1373808 for water samples, 9133–929157 for woodchip samples and 9776–34455 for root samples; see Table S1 for number of reads by sample). Reads for the duplicate woodchip and roots samples were averaged before further processing to provide more confidence in the identified genera. The abundance percentage of equal to or greater than 2% were termed as ”abundant” and displayed on the graphs, whereas the ratios smaller than 2% were added up and shown as a sum value called “other”. Reads unmatched to the Illumina version of the Greengenes database were categorized as “unclassified”. The statistical analyses, including the multivariate analysis on the CLPP results, as well as the heat maps on the genus abundances, were performed using XLSTAT software (©Addinsoft, New York, NY, USA).

## 3. Results and Discussion

### 3.1. Mixed Microbial Activities

**Figure 1:**
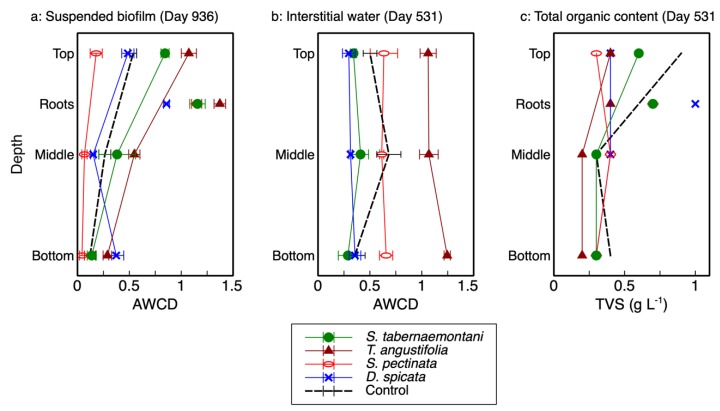
**a**: Microbial activities for biofilm from each bioreactor at 3 depths and plant roots. **b**: Microbial activities for interstitial water from each bioreactor at 3 depths (adapted from [1]). **c**: Total organic content of the suspended solution containing biofilm from wood-chip samples (top, middle and bottom) and plant roots (TVS: total volatile solids and AWCD: average well color development).

The AWCD—the average well color development in Biolog® plates and used as an indicator of microbial activity—and total richness for each reactor’s depth and plant root are presented in Table 1. Unlike the previously reported microbial activity in the interstitial water samples [2], which showed no consistent correlation with depth (Figure 1b), the results from the wood-chip samples showed a clear decreasing trend as the reactor depth increased (Figure 1a). As displayed in this figure, the activity of the microbial communities was highest at the top of each reactor (20-cm depth), decreased in the middle (40-cm depth) and, with the exception of *D. spicata*, decreased further at the bottom (60-cm depth). The highest activity was recorded for the *T. angustifolia* reactor, followed by *S. tabernaemontani*. Moreover, the rhizospheric activities were higher than those associated with the reactor substrate, where the highest rhizospheric activity pertained to *T. angustifolia*.

The two-way ANOVA on the wood-chip samples showed that both depth and vegetation were significant factors for the microbial activity (*p-value* < 0.0001). The significantly different activity at each depth indicated a distinct stratification through the vertical profile of each reactor. The pair-wise comparisons showed the activities were also significantly different between all the reactors except for the *D. spicata* and the unplanted units (*p-value* = 0.9). According to the interaction effect of vegetation and depth, the reactor planted with *T. angustifolia* showed significantly higher activities with respect to the control at all the three depths (*p-values* < 0.005), whereas *S. tabernaemontani’s* activities were significantly higher than control at the shallower depths (top and middle, *p-values* < 0.05). These differences were, however, consistently less prominent as the depth increased (Figure 1a).

The organic contents (TVS) of the detached biofilm from the top layer of *T. angustifolia*, *S. tabernaemontani* and control reactors were approximately two times higher than the middle and bottom layer samples (Figure 1c). The higher organic mass detached from the top layers of these reactors suggest that, compared to the lower layers, a more substantial biofilm was formed at the shallowest depth; thus, the higher observed activities of the better-established communities in the upper layers of these reactors were consistent with the biofilm mass.

The total richness (Table 1) showed a similar trend to the AWCD values, with a generally decreasing richness as the depth increased. Moreover, richness was closely correlated with AWCD values for wood-chip samples, as richness was lower for smaller catabolic activities. The declining richness in depth demonstrated a decrease in functional utility of the microbial community at greater depths [7]. This observation is in agreement with other studies (e.g., [7,9]).

**Figure 2:**
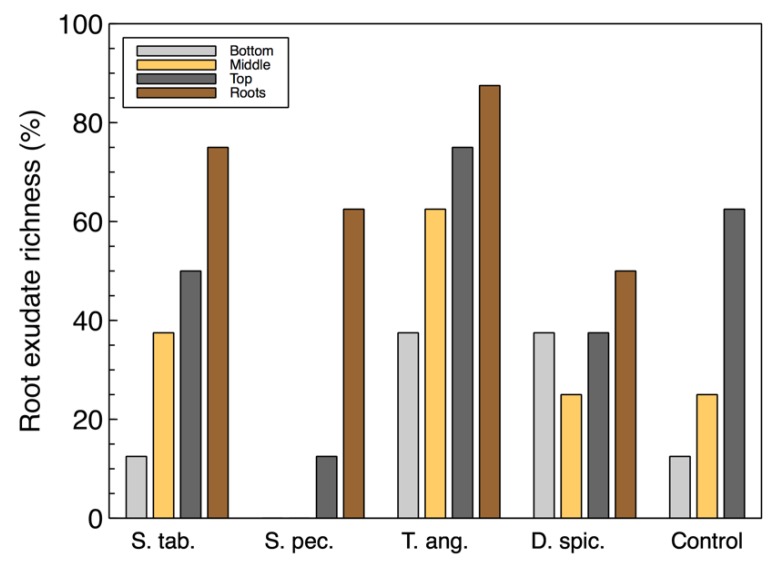
Microbial community richness of the biofilm samples based on the utilized carbon sources associated with plant root exudates.

For the most part, the richness of the utilized carbon sources associated with root exudates (Figure 2) were also similar to the total richness. However, the root exudate richness in the wood-chip samples from the unplanted reactor was found to be higher than some of the vegetated reactors. This suggests that although the control unit did not contain any plants, the microbial community was able to utilize these carbon sources and showed a richness of approximately 60%. This observation may also suggest that there is a link between the organisms capable of consuming root exudate sources and the colonized denitrifiers that were previously identified in the *T. angustifolia* and control units [2].

The higher activity in the reactors’ top layers and the greater rhizospheric activities compared to the activities associated with the wood chips alone were similar to the findings reported in treatment wetlands by Weber and Legge [7]. Zhao et al. [25] and Zhang et al. [26] also found that plants had a strong effect on the diversity and activity of the microbial communities in CWs. This greater microbial activity is likely due to the presence of the plant roots, which were established at a higher density in the top layer of the reactors. The roots may have carried over external microbial sources to the reactors at the time of plantation. The other important contribution of roots is their physical structure, which is adept at fostering microbial communities.

Additionally, the exudates from the plant roots are known to play an important role in the rhizosphere biology through the interactions with the soil and the microbial community [27]. Salomo et al. [9] reported that the decayed plant litter in the surface layer of their CW could be utilized by the microorganisms present in that layer. The authors also suggested that a lack of nutrient supply in deeper layers may have an impact on the carbon source utilization pattern of the microbial communities. In our system, however, the catabolic activities of the *T. angustifolia* and control reactors did not seem to suffer from the nitrate limitation that occurred in those units. Therefore, the combination of the potential external sources of microbes and physiochemical support of the root network, as well as the root exudates and presence of the plant litter were likely the main factors responsible for the higher activities and greater total richness in the *T. angustifolia* and *S. tabernaemontani* units.

It should, however, be noted that, compared with the unplanted unit, not all the plant species had a positive effect by increasing the microbial activity. The activity and richness of *D. spicata* and *S. pectinata* were often smaller than the control unit. These results suggest that the selection of a suitable plant species is an important factor for successful microbial development and growth and reactor treatment performance.

The AWCD of the biofilm can be compared with the AWCD of the interstitial water samples (Figure 1a,b). In general, the microbial activities of the top layers in the *T. angustifolia* and control reactors in the detached biofilm samples were similar to the average activities of the interstitial water samples in these two reactors. The other units did not show a consistent correlation between the two sampling methods. The biofilm extracted from the wood-chip substrate was able to provide a better picture of the developed stratification in the reactors. While the destructive sampling for biofilm extraction was conducted 405 days after the collection of interstitial water samples, the high microbial activities and denitrification rates implied that the *T. angustifolia* reactor had already acclimatized by the time water sampling was conducted. This indicates the stratified pattern of the microbial activities through depth was also likely present in the reactors when water sampling was performed. Thus, it was the interstitial water sampling method that failed to show the reactor’s stratification that was evident in the biofilm analysis.

The lack of a recognizable trend from the water sampling method was likely due to the fact that some gravity-driven mixing of the vertical water flows was expected at the time of sampling. This could prevent each sample from being a perfect representation of the stagnant water phase adjacent to the biofilm. Furthermore, due to the unknown spacing between the tip of the sampling tubes buried inside the media and the coarse wood-chip grains, the characteristics of the collected water samples could differ between the sampling locations in one reactor and across all the reactors. That is, the buried end of one internal sampling tube could be in direct contact with a wood-chip grain, while another tube could be positioned a few millimeters away from the surface of a wood chip where biofilm would be formed. Therefore, the interstitial water sampling method did not seem to demonstrate the differences between the microbial activities along the reactors’ depth as well as the biofilm sampling did.

A total of five (Figure S1a) and four (Figure S1b) groups were identified based on the similarities of the data in multivariate analyses for the biofilm samples (PCA and AHC plots). The top layer sample of the wood-chip media in the *T. angustifolia* reactor was grouped with all the plant roots, which is in agreement with the generally higher activities of these samples as reported in Figure 1. The media samples in the top two layers of the *S. tabernaemontani* reactor and the top layer of the unplanted and the middle layer of *T. angustifolia* and control reactors were also grouped together. Although subtle, it seems that the similarity and dissimilarity of the biofilm samples are more influenced by different depths, rather than the planted species. This is in contrast to the similar analysis on the interstitial water samples, as shown in Figure S2. In the interstitial water method, the grouping of the *T. angustifolia*, *S. tabernaemontani* and *D. spicata* samples was more distinctly influenced by their respective plant species. 

### 3.2. Denitrifying Gene Quantities

**Figure 3:**
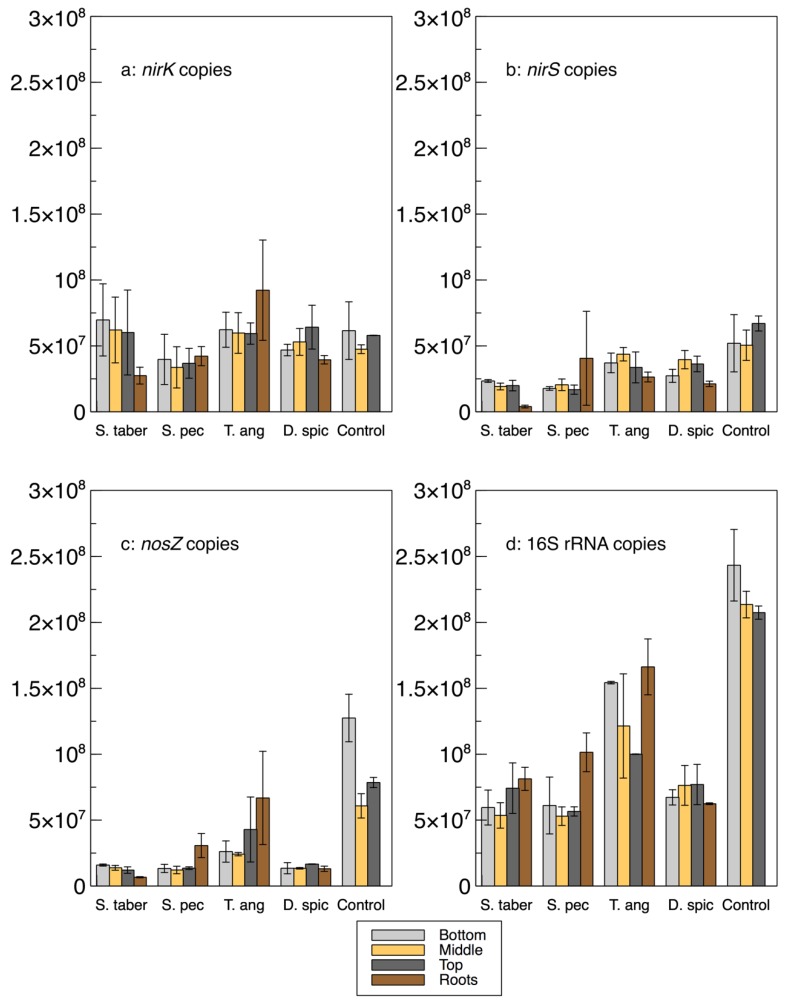
Gene copy numbers (a: *nirK*, b: *nirS*, c: *nosZ* and d: 16S rRNA) in the extracted biofilm from the bioreactors’ media at 3 depths and in plant roots. Error bars: standard deviations.

A relative qPCR method was used to quantify the abundance of the microbes encoding the *nirK*, *nirS*, *nosZ* and 16S rRNA genes (Figure 3 and Table 2). As such, it is important to note that while the calculated gene copies provide important information about the bacterial communities and their abundance in relation to the other samples and genes, these values are not absolute numbers and, thus, cannot be directly compared with the gene copy numbers in the literature. 

The abundance of the 16S rRNA gene was higher in the control and *T. angustifolia* reactors, while *T. angustifolia*’s roots showed the greatest abundance of 16S rRNA among the roots. Since the reactors were not augmented with an external source of bacteria, the total bacterial DNA (16S rRNA) abundance can be used as a general indicator for the natural development of the bacterial population in each reactor. The higher 16S rRNA count in these two reactors was in good agreement with their high denitrification performance at the time of the core sampling [2]. Unlike the mixed community results (Figure 1a), the 16S rRNA copies in these two reactors increased through the reactor depth, demonstrating the highest abundance in the bottom layer of the wood-chip media. The other reactors did not display a consistent pattern regarding their 16S rRNA copies at different depths. Except for the *D. spicata* reactor, which was previously found to have the lowest microbial activity via CLPP (Figure 1a), the abundance of 16S rRNA in the root samples of each reactor was higher than the reactor’s respective wood-chip samples, indicating a generally greater potential for the presence of the bacterial communities in the plants’ rhizosphere.

**Figure 4:**
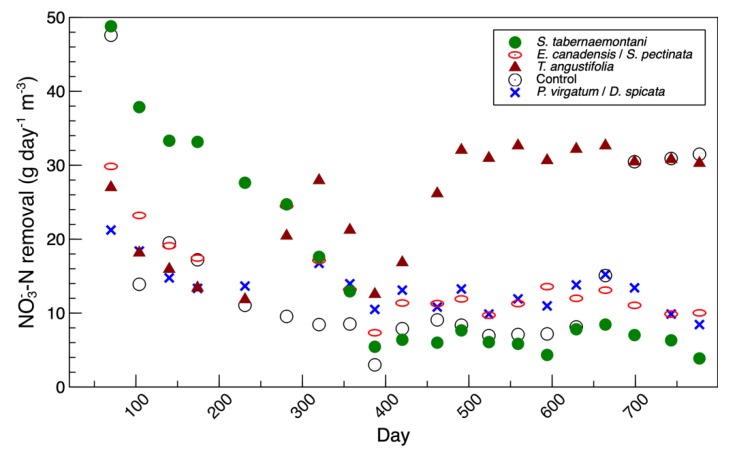
Temporal trend of nitrate mass removal rates.

The average *nirK* copies in the control reactor’s media was similar to the *nirS* genes in this reactor, whereas *nirK* copies were mostly higher than *nirS* in the other units. The *nirK* gene copies did not seem to reflect the nitrate removal performance of the reactors, shown in Figure 4 and reported in Fatehi-Pouladi et al. [2]. For instance, the *S. tabernaemontani* reactor that showed a decreased denitrification efficiency towards the end of the experiment was found to be the unit with the highest *nirK* copies. These results are in agreement with our earlier finding, where we reported the *nirK* fold-changes in the reactors’ interstitial water were negligible [2]. In the current paper, although *nirK* was found in abundance and was typically greater than *nirS* (*nirK*/*nirS* of between one to seven), its direct role in complete denitrification was less significant. This may suggest that the bacteria containing *nirK* were present but may not have been expressing *nirK* as much as the other nitrite reductase gene, *nirS,* was expressed. As well, some of the denitrifying genes, including *nirK,* are also present in bacteria involved in other biological nitrogen transformation pathways, such as incomplete denitrification and dissimilatory nitrate reduction to ammonia (DNRA) [5].

The abundance of the *nirS* gene was more consistent with the water quality results, as the control and *T. angustifolia* reactors contained the highest *nirS* counts, while the lowest *nirS* abundance was found in the *S. tabernaemontani*’s roots and wood-chip media. The differences between the *nirS* gene copies among the reactors were, however, small compared to the *nosZ* gene, whose average highest abundance in the control reactor was almost seven times higher than that of the lowest copies in the *S. pectinata* unit. Although the cytochrome enzyme encoded by *nirS* and the Cu-containing enzyme encoded by *nirK* are needed to catalyze the reduction of nitrite to nitric oxide, the *nosZ* enzyme is responsible for the last step of the denitrification pathway by reducing nitrous oxide [28]. As such, the copy number of the bacteria with the *nosZ* gene is an important indicator for a potentially complete denitrification pathway. The *nirS* and *nosZ* genes in our study appeared to be the key gene markers for the observed denitrification, whose populations were positively correlated with the nitrate reduction efficiencies.

Various proportions of the denitrifying genes have been reported in the literature. Warneke et al. [29] found a *nirK*/*nirS* ratio of between 0.1 to 1.0 in their denitrification bed samples, and Ligi et al. [24] reported a range of 0.07 to 0.74 for their wetland soils. These values were smaller than our ratios of 1.0 and up to 3.1 in the wood chips. Nonetheless, the reported *nosZ*/Σ*nir* ratios of 0.1 to 0.7 [29] and 0.04 to 0.40 [24] were in agreement with our wood-chip *nosZ*/Σ*nir* ratios of 0.2 to 0.8. The *nirK*%, *nirS*% and *nosZ*% in Ligi et al. [24] were generally much smaller than our values. However, given that the authors’ soil samples were from a river wetland area, a substantially different composition of bacterial community from that of our study is expected. Since our reactors were operated in a more isolated environment and were specifically designed to promote denitrification, it was anticipated that a higher ratio of the total bacterial population would express nitrite and nitrous oxide reductase genes. A similar observation was reported in Warneke et al. [29], where *nirK*%, *nirS*% and *nosZ*% ratios in multiple carbon-based denitrification barrels were higher than those reported in Ligi et al. [24] for a potentially more complex wetland system.

Among the plant roots, *T. angustifolia* stood out by demonstrating the greatest copy numbers of *nirK*, *nosZ*, 16S rRNA, and *nir*+*nosZ*, as well as the ratios of *nosZ*% and *nosZ*/Σnir. These observations confirm the previously discussed important role of this species in the denitrifying bioreactors and further support that the maximum denitrification in this reactor, which occurred faster than the other reactors and benefited from its rhizosphere and its rich bacterial genome.

In general, and similar to the interstitial water samples, a consistent correlation between the denitrifying gene copies and reactor depth could not be established. Although the *T. angustifolia* and control reactors showed an inverse correlation between the 16S rRNA copies and the reactor depth, no consistent pattern could be identified for the other genes or in the other reactors. The decreased 16S rRNA copies in the *T. angustifolia* and control units in the lower depths could be due to the lack of substrates, particularly nitrate, in the layers closer to the reactor’s bottom.

Both interstitial water and biofilm sampling methods provided useful information regarding the potential activities and quantification of the denitrifying bacteria. The high gene abundance and gene fold-change in the reactors with high denitrification efficiency were recorded using the water and biofilm sampling methods, respectively. In the future, it is recommended to analyze these target genes using the absolute qPCR method, in which the standard curve is created by cloning known gene concentrations into plasmids. This method would further help to analyze the natural development of denitrifying bacteria and compare the expression levels with those of the other environmental samples.

### 3.3. 16S rRNA Amplicon Sequencing—Interstitial Water

The temporal changes of the bacterial composition in the interstitial water samples from the *S. tabernaemontani*, *T. angustifolia* and unplanted reactors are presented in Figures SI3–SI6. In the *S. tabernaemontani* reactor, the total abundance of the classified genera was highest in the beginning of the experiment (day 147, Figure S3). This was consistent with the denitrification performance of this reactor, which was at its peak during the beginning phase of the study (Figure 4). The *Bacillus* genera had the highest abundance in this sampling event. It has been reported that denitrification is a common feature of *Bacillus* spp. [30], and the species related to this genus are among the most common denitrifying bacteria [31]. The other abundant genera on day 147 were *Clostridium*, *Leuconostoc*, *Rhodococcus* and *Longilinea*, which were not greater than 2% during the later time points. The dominant classified genus in the rest of the water samples from *S. tabernaemontani* was *Geobacillus*, which had an abundance of less than 1% on day 147 but were consistently found in other sampling events (maximum of 13.3% on day 389). After *Geobacillus*, the communities related to genera *Pseudoalteromonas* and *Deinococcus* were among the abundant groups between days 359 and 517, while *Weissella* was the second-most abundant genus on the final sampling day (day 665). Compared to the samples collected approximately one year after the project start-up (days 359 to 665), the distinct structural composition of the water samples on day 147 indicated that these different bacterial communities played an important role in the denitrification that occurred early in this reactor.

A clear shift in the bacterial composition of the water samples in the *T. angustifolia* reactor was found between days 442 and 517 (Figure S4). This was consistent with the maximum nitrate reduction, which was achieved near day 500 (Figure 4). Similar to the *S. tabernaemontani* reactor, *Geobacillus* was the most abundant genus in the *T. angustifolia* reactor before day 516, but the major communities present in the second phase were *Sulfurimonas* (max: 24.8%), *Sulfurospirillum* (max: 8.0%), *Bacillus* (max: 11.6%) and *Thauera* (max: 18.6%). In addition, the final samples on day 665 contained abundant *Thermogemmatispora* (max: 8.7%) and *Geobacter* (max: 11.2%) spp. *Sulfurospirillum* and *Thauera* are both nitrate-reducing bacteria [32,33]. *Sulfurimonas paralvinellae*, one of the species prevalent in this reactor (Figure S5), is a facultatively anaerobic chemolithoautotroph, sulfur- and thiosulfate-oxidizing bacteria, which can use nitrate or ammonium as its nitrogen source [34]. The sulfate reduction that was observed in this reactor between days 500 and 700 (data not shown) can be explained by the sulfate-reducing bacteria (SRB), such as species affiliated with the genus *Desulfotomaculum* [35]. These organisms were found among the abundant species on day 601 (*Desulfotomaculum acetoxidans* at approximately 2.3%) but decreased to an average of 0.8% on day 665. Nonetheless, the well-studied sulfate-reducing bacteria from the genus *Desulfovibrio* and order *Desulfovibrionales* [36] were less than 0.37% during the entire operation.

The most abundant species in the water samples from the *T. angustifolia* reactor from day 517 onwards included *Thauera aromatica* (max: 12.6% on day 517), *Bacillus ginsenggisoli* (max: 8.6% on day 601), *Sulfurimonas paralvinellae* (max: 2.7% on day 517) and *Thermogemmatispora foliorum* and *Geobacter lovleyi* (max: 8.6% and 6.3% on day 665).

In the unplanted reactor, the colonies affiliated with the genus *Mycoplasma* were present in most of the samples (max: 14.2%), although the ratio demonstrated a decreasing trend toward day 665 (Figure S6). 

The abundant species from this genus was *Mycoplasma insons*, which was found between 0.2% and 13.2% (data not shown). The other main abundant genera in this reactor were *Geobacillus* (max: 5.8%) and *Alkalibacterium* (max: 5.1%). The *Geobacillus* spp. are of interest in wood-chip bioreactors, since they comprise a group of bacteria that include denitrifiers and are known for their potential for degradation of hemicellulose and lignocellulose biomass, as well as their application in lignocellulosic biofuel production [37,38]. *Bacillus* spp. are similarly capable of utilizing alkaline lignin as a carbon or energy source [39]. These species may have contributed to the breakdown of the organic matter from wood chips that was quantified by the high outflow five-day biochemical oxygen demand (BOD_5_) and chemical oxygen demand (COD) in the *T. angustifolia* and unplanted reactors (refer to [2] for more details).

The influent sample was expectedly less diverse than the wood-chip bioreactors (Figure S7). There were only two abundant genera with a ratio of greater than or equal to 2%, namely *Burkholderia* (27%) and *Salinispora* (31%), while the most abundant species was *Salinispora tropica* (30.1%). *Salinispora* spp. are the bacteria originating from a saline habitat and require seawater or sodium for growth [40]. Thus, it is not surprising that these organisms were established in our synthetic greenhouse effluent with high salinity.

### 3.4. 16S rRNA Amplicon Sequencing—Biofilm

**Figure 5:**
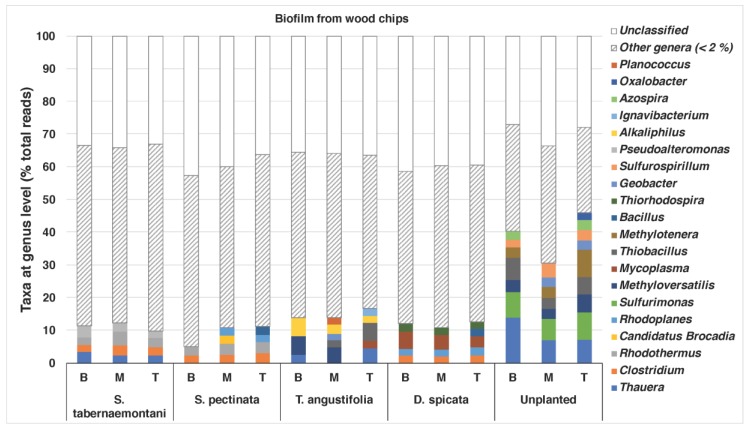
Relative bacterial composition at the genus level in the biofilm wood-chip samples. ”Other genera” is the sum ratios of genera with less than 2% abundance. B, M and T represent the bottom (60 cm), middle (40 cm) and top (20 cm) depths, respectively.

The bacterial compositions of the biofilm that was extracted from the wood chips are shown in Figure 5. The unplanted reactor exhibited the most diverse structure of the genera, with abundances of >/= 2%. This reactor showed the highest nitrate and sulfate reductions among the other units near the end of the experiment when the core sampling was performed (data for sulfate reduction not shown). *Thauera* (max: 13.9%), *Sulfurimonas* (max: 8.3%), *Methyloversatilis* (max: 5.6%), *Thiobacillus* (max: 6.8%) and *Methylotenera* (max: 8.4%) were among the identified genera in this reactor. The genera *Thauera*, *Methyloversatilis* and *Thiobacillus* were also found in the wood chips from the *T. angustifolia* reactor (max: 4.5%, 5.6% and 5.4%, respectively), which achieved a similar high denitrification and sulfate reduction earlier than the unplanted reactor. *Methyloversatilis* has been shown to have denitrification capacity by reducing nitrate to nitrite [41], and *Thauera* spp. have been associated with denitrifying communities [5]. The *T. angustifolia* reactor was also the only unit that had abundant *Alkaliphilus* spp., while the *S. tabernaemontani*, *S. pectinata* and *D. spicata* had between 2% and 3% of the genus *Clostridium*.

**Figure 6:**
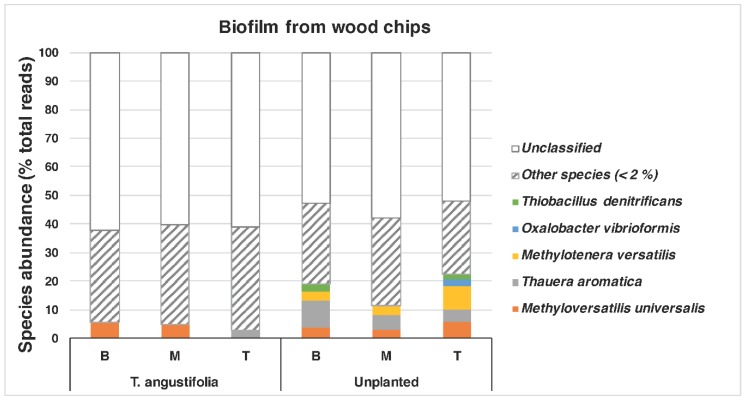
Relative bacterial composition at the species level in the *T. angustifolia* and unplanted reactors’ media. ”Other species” is the sum ratios of species with less than 2% abundance. B, M and T represent the bottom (60 cm), middle (40 cm) and top (20 cm) depths, respectively.

A closer look at the species found in the *T. angustifolia* and unplanted reactors revealed that *Thauera aromatica* (max: 9.5%) and *Methyloversatilis universalis* (max: 5.6%) were among the dominant species in the media of these reactors, whereas *Methylotenera versatilis* was mostly found only in the unplanted reactor (max: 8.4%) (Figure 6).

**Figure 7:**
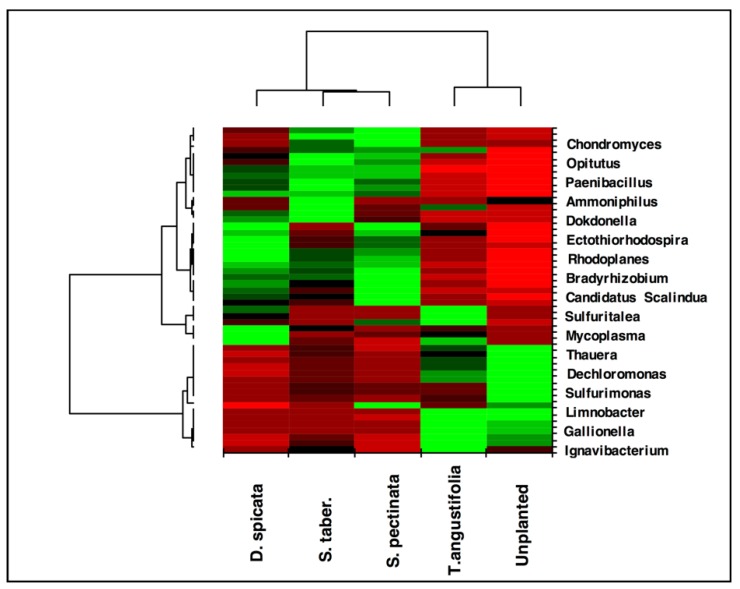
Heatmap and dendrogram of the biofilm from wood chips, based on the abundance of the microbial community at the genus level. Average ratios of 3 depths for each reactor and a nonspecific filtering for interquartile range < 0.25 were used. Only the main genera are shown for clarity and to illustrate the overall similarity between reactors.

The heat map created for the taxa ratios at the genus level demonstrated high similarity between the *T. angustifolia* and unplanted reactors (Figure 7). This grouping indicates that these two reactors developed a generally similar microbial community at the end of the experiment, and the populations related to the genera found in higher abundance in these reactors were responsible for the similar treatment performance observed in these units.

The genera found in the root samples were generally less diverse compared to the water and wood-chip samples (Figure S9a). The organisms affiliated with *Plesiomonas* and *Tolumonas* were found in the roots of *S. tabernaemontani* (13.3% and 10.0%, respectively), whereas *Azospira* (13.8%) was found abundant in the *S. pectinata* roots. The *T. angustifolia* roots had organisms related to *Mycoplasma* (2.3%), *Thauera* (2.3%) and *Azospirillum* (2.1%), while all of the genera in the roots of *D. spicata* were less than 2%. *Azospira restricta* (5.6%) and *Tolumonas auensis* (9.9%) were the dominant species in the *T. angustifolia* and *D. spicata* roots, respectively (Figure S9b).

In comparison with the media samples, the heatmap for the root samples demonstrated more individual groups, as *S. pectinata* and *D. spicata* were shown to be more similar to one another, while *S. tabernaemontani* was different from the other three plants (Figure S8).

Lu et al. [5] have reviewed the microbial ecology of denitrification in wastewater systems and have discussed the common denitrifier genera that are found in denitrifying reactors. Based on the information provided by the authors, we selected the following genera as those potentially responsible for denitrification in our reactors and calculated the sum of the individual ratios from their taxa information. These selected populations included *Hyphomicrobium*, *Paracoccus*, *Azoarcus*, *Thauera*, *Methylophaga*, *Pseudomonas*, *Acidovorax*, *Methyloversatilis*, *Thiobacillus* and *Comamonas*.

**Figure 8:**
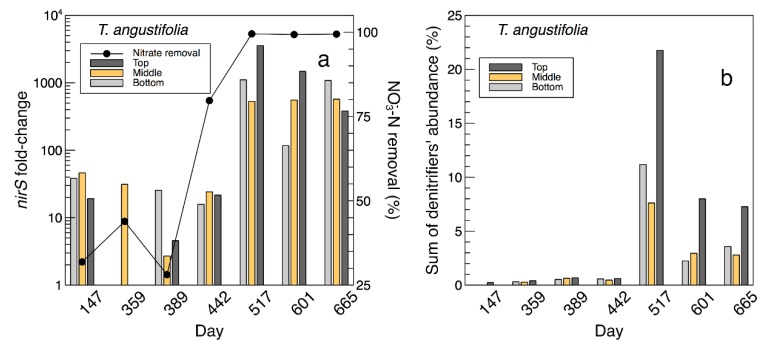
**a**: Temporal *nirS* fold-change in *T. angustifolia* bioreactor at 3 depths and reactor’s nitrate removal rate (derived from [2]). **b**: Temporal change of the sum of abundance of common denitrifiers in the *T. angustifolia* reactor.

The fold-changes of the expressed *nirS* gene and nitrate removal efficiencies are presented in Figure 8a [2], while the sum of the denitrifier genera found in the water samples from the *T. angustifolia* reactor and their change during the operation time are shown in Figure 8b. These figures demonstrate the positive correlation between the higher denitrifier abundance and the greater *nirS* populations on day 517 and their gradual decrease to the end of the operation. In both figures, the water samples from the top depth demonstrated larger populations of denitrifiers and those expressing the *nirS* gene on days 517 and 601.

**Figure 9:**
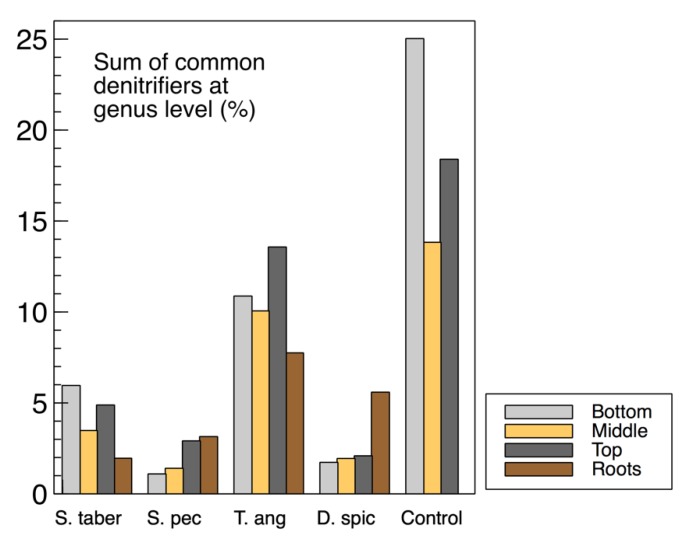
Abundance of denitrifiers in the wood-chip and root samples.

The sum of common denitrifiers in the biofilm samples from the media (wood chips) and plant roots are displayed in Figure 9, while the *nirS*, *nosZ* and 16S rRNA genes were discussed earlier (Figure 3). Some similarities were observed between the biofilm denitrifier populations and those expressing the *nosZ* gene. In both methods, the *S. tabernaemontani* and *T. angustifolia* roots contained the smallest and largest denitrifier populations among the root samples, respectively. Moreover, the media samples from the control reactor showed the greatest abundance, followed by the *T. angustifolia* unit. As well, the patterns of the two bioreactors for *nosZ* copies through the reactor depth were similar to the denitrifier abundance, showing the greatest copies and abundance at the top depth in the *T. angustifolia* reactor and at the bottom depth for the unplanted unit. 

As mentioned earlier, the higher denitrifier ratio in the *T. angustifolia* bioreactor can be attributed to the contributions of the plant’s rhizosphere and the root exudates. Nonetheless, unlike the mixed microbial activity results for the wood-chip media (Figure 1a), a consistent trend of denitrifier abundance through reactor depth was not found in all the bioreactors. This finding indicates that, although the rhizosphere of *T. angustifolia* likely had an important effect on the higher *nosZ* copies and denitrifier population at the shallowest depth, the other plants did not provide a similar effect in their respective reactors. Furthermore, as the consistently greater activities that were observed at the top depth in these reactors did not correlate with the abundance of the classified organisms via 16S amplicon sequencing, these higher mixed microbial activities may have been caused by other microbial communities in these open reactors.

**Figure 10:**
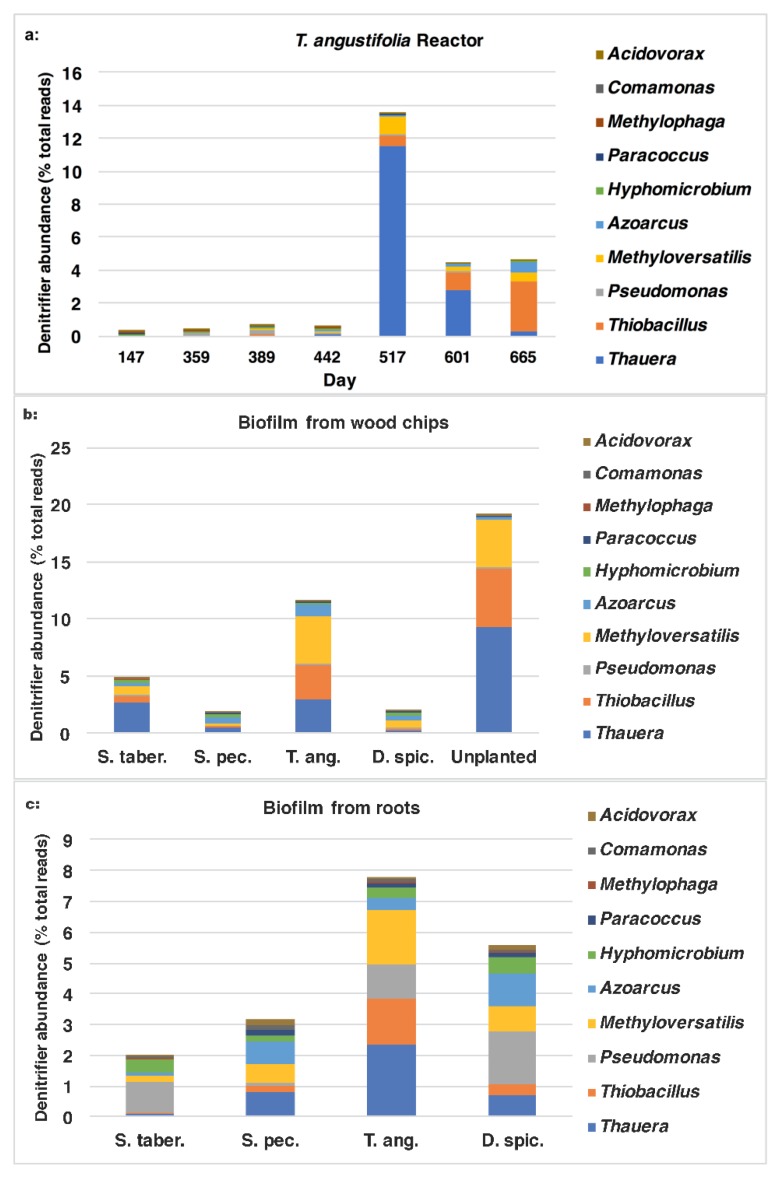
Abundance of denitrifiers at genus level in **a**: *T. angustifolia* bioreactor’s water samples, **b**: biofilm from wood chips and **c**: biofilm from plant roots.

In addition to the sum quantity of the denitrifier populations comprised of the 10 genera mentioned earlier, it is of interest to investigate which microorganisms were found in greater proportions and had a larger contribution to the overall performance of the bioreactors. The ratios of each individual genus are demonstrated in Figure 10a–c. In the water samples from the *T. angustifolia* reactor (Figure 10a) on day 517, the major organisms belonged to *Thauera* (11.6%) and, to a lesser extent, *Methyloversatilis* (1.1%), *Thiobacillus* (0.6%) and *Azoarcus* (0.1%). As the operation progressed, the communities affiliated with *Thauera* and *Methyloversatilis* decreased to 0.3% and 0.5%, respectively, while *Thiobacillus* and *Azoarcus* increased to 3.0% and 0.7%, respectively.

For the wood-chip samples (Figure 10b), the majority of the organisms in the two bioreactors with the highest nitrate-reduction performances were affiliated with *Thauera*, *Thiobacillus* and *Methyloversatilis* and, to a smaller degree, *Azoarcus*. The abundance of *Thauera* and *Thiobacillus* were higher for the unplanted reactor’s media compared to the *T. angustifolia* unit (9.3% vs. 3.0% and 5.1% vs. 3.0% respectively).

Similarly, *Thauera* (2.3%), *Thiobacillus* (1.8%) and *Thiobacillus* (1.5%) were the major genera in the biofilm detached from the *T. angustifolia* roots (Figure 10c). The populations related to *Pseudomonas* were another member of the denitrifiers that were found as the dominant denitrifier genus in the *S. tabernaemontani* (1.0%) and *D. spicata* (1.7%) roots.

These results demonstrate the importance of the populations associated with the genera *Thauera*, *Thiobacillus* and *Methyloversatilis*, as these were the predominant denitrifying communities. Moreover, *Sulfurimonas*, *Sulfurospirillum*, *Methylotenera*, *Bacillus* and *Geobacter* were the other general abundant genera that formed the community structure of the reactors with high denitrification performance. *Thiobacillus* and *Sulfurimonas* were the most commonly reported sulfur-oxidizing autotrophic denitrifiers, and *Methyloversatilis*, *Thauera*, *Thiobacillus* and *Geobacter* were found as the dominant genera in systems where methanol, ethanol or sulfur were the electron donors in the denitrification process [42]. These findings suggest that both autotrophic denitrification pathways through organisms related to *Thiobacillus* and those belonging to *Sulfurimonas* (based on sulfur oxidation), as well as heterotrophic denitrification facilitated by *Thauera* and methylotrophs such as *Methyloversatilis* spp., may have played significant roles in our denitrifying bioreactors.

Moreover, *Sulfurospirillum* spp. are known to be versatile and are able to grow organotrophically and chemolithotrophically in environments where organic and inorganic electron donors are present [32]. The presence of organisms related to *Sulfurospirillum,* which are known for their ability to reduce sulfur [43,44], as well as sulfate-reducing bacteria such as *Desulfotomaculum* spp. [35] and species affiliated with *Sulfurimonas* with sulfur- and thiosulfate-oxidizing capacity [34], indicate the development of a rich and complex sulfur cycle in our bioreactors.

Furthermore, the methylotrophic populations related to *Methyloversatilis* and *Hyphomicrobium* spp. were reported in denitrifying reactors metabolizing methanol [45]. Methylotrophic bacteria are capable of utilizing methanol or methane as their source of energy and, therefore, can potentially reduce the level of the greenhouse gas emissions [46]. The production of methane in wood-based denitrifying bioreactors has been previously reported (e.g., [29,47]). The microbial community sequencing results in this study indicate that, although methane may have been produced via the breakdown of the organic carbon in the wood biomass [48], the types of the abundant bacteria developed in the reactors were capable of utilizing some of the produced methane as a source of energy for nitrate reduction.

This study demonstrated the superior role of *T. angustifolia* in comparison with the other plant species to enhance the biological processes and facilitate denitrification in a shorter period of time. However, it was noted that the unplanted reactor was also capable of hosting a diverse population of microorganisms, including common denitrifiers, many of which were similar to those found in the bacterial composition of the *T. angustifolia* reactor. Therefore, the presence of populations with the capability to decompose wood products (such as *Geobacillus* spp.) may be an equally important factor in successful denitrification in wood-based systems. The fungi species are also known to contribute to denitrification directly [49] or by degrading wood compounds and facilitating bacterial denitrification [50,51]. The interaction between the wood-decaying fungi and the bacteria found in wood is, however, poorly understood [52]. As such, it is recommended to further investigate the presence and function of the fungi species in wood-chip bioreactors in order to better understand how these two communities originating from different phyla may aid denitrification by causing wood degradation or other potential mechanisms.

## 4. Conclusions

The high nitrate reduction reported in the wood-chip bioreactor planted with *T. angustifolia* and the unplanted reactor was consistent with the higher microbial activities and denitrifier populations. As well, the denitrification performance in these units correlated with a distinct shift in the phylogenetic composition and structure of the microbial community in the extracted water and biofilm samples.

While for most of the bioreactors, the biofilm detached from the roots demonstrated higher microbial activities and greater 16S rRNA copies than the wood-chip samples, the abundance of the denitrifying communities were not necessarily higher in the roots. The rhizosphere of *T. angustifolia*, however, displayed higher microbial activities than the other plant species. The rhizosphere of this plant and other factors, such as the presence of bacteria capable of wood decomposition, were discussed as the facilitator of the biological denitrification in our wood-based systems.

The relationship between the microbial potential function (bacteria containing denitrifying genes) and the microbial structure (denitrifier communities) was also analyzed. *Thauera*, *Thiobacillus* and *Methyloversatilis* were the most prevalent genera among the group of denitrifiers, while *Sulfurimonas*, *Sulfurospirillum*, *Methylotenera*, *Bacillus* and *Geobacter* were the other abundant genera in the bioreactors. The potential role of fungi and their interaction with the wood-dwelling bacteria were recommended to be investigated further in future studies.

The results of this study showed *T. angustifolia* is a suitable plant species for application in denitrifying treatment systems. This species was successful in establishing microbial communities in a shorter period than the unplanted unit and performed quite well in the treatment task.

## Figures and Tables

**Table 1:** Mean average well color development (AWCD) values’ standard deviation and mean total richness (%) in each bioreactor at three depths (wood chips) and plant roots. Note that plant roots were not subject to pair-wise comparisons.

Reactor	Top		Middle		Bottom		Roots	
	AWCD ^A^	Rich. (%)	AWCD ^B^	Rich. (%)	AWCD ^C^	Rich. (%)	AWCD	Rich. (%)
Control ^a^	0.54 ± 0.03	52	0.26 ± 0.06	26	0.12 ± 0.05	16	-	
*S. tabernaemontani*^b,^*	0.84 ± 0.04	55	0.38 ± 0.18	35	0.14 ± 0.04	19	1.16 ± 0.07	81
*T. angustifolia*^c,^*	1.07 ± 0.07	71	0.55 ± 0.05	48	0.29 ± 0.04	39	1.37 ± 0.06	87
*S. pectinata*^d,^*	0.18 ± 0.06	23	0.07 ± 0.03	3	0.04 ± 0.02	10	1.15 ± 0.05	68
*D. spicata* ^a^	0.49 ± 0.06	42	0.15 ± 0.02	19	0.37 ± 0.07	32	0.86 ± 0.03	71

* Data with significant difference from the control reactor (*p-value* < 0.05). Different letters: significant paired difference among reactors (lowercase) and depths (uppercase) (*p-value* < 0.05).

**Table 2:** Functional denitrifying gene parameters ± (SD) in the biofilm from the wood-chip samples (average of 3 depths), as well as the root samples.

Genes	Unit	Control	*S. tabernaemontani*		*T. angustifolia*		*S. pectinata*		*D. spicata*	
		*Wood Chips*	*Wood Chips*	*Roots*	*Wood Chips*	*Roots*	*Wood Chips*	*Roots*	*Wood Chips*	*Roots*
*nirS*	copies	5.7 × 10^7^ (9.1 × 10^6^)	2.1 × 10^7^ (2.3 × 10^6^)	4.0 × 10^6^	3.8 × 10^7^ (5.1 × 10^6^)	2.6 × 10^7^	1.8 × 10^7^ (1.9 × 10^6^)	4.1 × 10^7^	3.4 × 10^7^ (6.4 × 10^6^)	2.1 × 10^7^
*nirK*		5.6 × 10^7^ (7.3 × 10^6^)	6.4 × 10^7^ (5.1 × 10^6^)	2.7 × 10^7^	6.0 × 10^7^ (1.6 × 10^6^)	9.2 × 10^7^	3.7 × 10^7^ (3.0 × 10^6^)	4.2 × 10^7^	5.5 × 10^7^ (8.8 × 10^6^)	3.9 × 10^7^
*nosZ*		8.9 × 10^7^ (3.5 × 10^7^)	1.4 × 10^7^ (1.9 × 10^6^)	6.7 × 10^6^	3.1 × 10^7^ (1.0 × 10^7^)	6.7 × 10^7^	1.3 × 10^7^ (7.5 × 10^5^)	3.1 × 10^7^	1.5 × 10^7^ (1.8 × 10^6^)	1.3 × 10^7^
16S		2.2 × 10^8^ (1.9 × 10^7^)	6.2 × 10^7^ (1.1 × 10^7^)	8.1 × 10^7^	1.3 × 10^8^ (2.7 × 10^7^)	1.7 × 10^8^	5.7 × 10^7^ (4.0 × 10^6^)	1.0 × 10^8^	7.4 × 10^7^ (5.4 × 10^6^)	6.2 × 10^7^
Σ*nir* + *nosZ* ^1^		2.0 × 10^8^ (4.1 × 10^7^)	9.9 × 10^7^ (9.0 × 10^6^)	3.8 × 10^7^	1.3 × 10^8^ (5.5 × 10^6^)	1.9 × 10^8^	6.8 × 10^7^ (2.4 × 10^6^)	1.1 × 10^8^	1.0 × 10^8^ (1.5 × 10^7^)	7.4 × 10^7^
*nirS*% ^2^	%	25.8 (5.8)	34.0 (6.4)	4.9	31.2 (6.3)	15.9	32.4 (5.4)	40.0	46.5 (5.7)	34.0
*nirK*% ^2^		25.2 (2.9)	104.7 (20.5)	33.7	49.6 (9.5)	55.5	64.6 (0.8)	41.6	74.2 (8.0)	63.2
*nosZ*% ^2^		39.6 (12.0)	22.9 (5.8)	8.2	26.6 (14.2)	40.2	22.9 (1.1)	30.3	19.8 (2.0)	21.0
*nirK*/*nirS*	-	1.0 (0.2)	3.1 (0.1)	6.9	1.6 (0.2)	3.5	2.0 (0.3)	1.0	1.6 (0.2)	1.9
*nosZ*/Σ*nir*	-	0.8 (0.3)	0.2 (0.0)	0.1	0.3 (0.1)	0.6	0.2 (0.0)	0.4	0.2 (0.0)	0.2

^1^ Sum of *nirK*, *nirS* and *nosZ* gene copies. ^2^ Ratio of a target functional gene over the total 16S rRNA quantity, expressed in percentage.

## Supplementary Materials

The following are available online at https://www.mdpi.com/2223-7747/9/3/289/s1, Figure S1: Multivariate analysis for wood-chip and root samples based on the carbon utilization patterns (CSUP). Figure S2: Multivariate analysis for the interstitial water samples based on the carbon utilization patterns (CSUP). Figure S3: Temporal change of relative bacterial composition at genus level in the *S. tabernaemontani* reactor’s interstitial water samples. Figure S4: Temporal change of relative bacterial composition at the genus level in the *T. angustifolia* reactor’s interstitial water samples. Figure S5: Temporal change of relative bacterial composition at the species level in the *T. angustifolia* reactor’s interstitial water samples. Figure S6: Temporal change of relative bacterial composition at the genus level in the unplanted reactor’s interstitial water samples. Figure S7: Relative bacterial composition at the genus level in the influent sample collected on Day 442. Figure S8: Heatmap and dendrogram of the biofilm from roots, based on the abundance of the microbial community at the genus level. Figure S9: Relative bacterial composition at a: the genus level, and b: at the species level in the biofilm root samples. Table S1: The total number of reads per sample included in this study. Woodchip and root samples were taken in duplicate and averaged.
