# Supplementary material for: Influence of Plant Species on Microbial Activity and Denitrifier Population Development in Vegetated Denitrifying Wood-Chip Bioreactors
Source: Plants (Basel). 2020 Feb 26;9(3):289. doi: 10.3390/plants9030289 (PMC7154878; doi:10.3390/plants9030289)
Supplement: Supplementary file 1 [file plants-09-00289-s001.pdf]

Supplementary Materials

Materials and Methods (16S rRNA amplicon sequencing)

First, to amplify 16S rRNA, 12.5 ng of microbial genomic DNA, 1 μM of each forward (5′-TCGTCGGCAGCGTCAGATGTGTATAAGAGACAGCCTACGGGNGGCWGCAG-3′) and reverse (5′-GTCTCGTGGGCTCGGAGATGTGTATAAGAGACAGGACTACHVGGGTATCTAATCC-3′) primers, and 12.5 μl of NEBNext Q5 HotStart HiFi PCR Master Mix (New England Biolabs Ltd., Whitby, ON, Canada) were used in a polymerase chain reaction (PCR) on a T100 Thermal Cycler (Bio-Rad Laboratories Ltd., Mississauga, ON, Canada) with the following program: 95 °C for 3 min, 25 cycles of 95 °C for 30 s, 55 °C for 30 s, and 72 °C for 30 s, with a final step of 72 °C for 5 min. To purify the 16S fragment, Agencourt AMPure XP beads (Beckman Coulter Inc., Mississauga, ON, Canada) were used and 80% ethanol washes were conducted. Second, 5 μl of the purified 16S amplicon product was added to 25 μl NEBNext Q5 HotStart HiFi PCR Master Mix (New England Biolabs Ltd., Whitby, ON, Canada), and 5 μl of each Nextera XT Index 1 and 2 Primers Set A (Illumina Canada Ulc., Victoria, BC, Canada) to add a unique barcode and a sequencing adapter to each sample and bead purification as mentioned above. The concentration of each sample library was measured on the Qubit fluorometer with a dsDNA HS assay kit (Invitrogen, Ottawa, ON, Canada).

Table S1. The total number of reads per sample included in this study. Woodchip and root samples were taken in duplicate and averaged.

Samples	Sample	Total number of reads
T. angustifolia Water Samples	TY100	469173
	TY86	522852
	TY85	930731
	TY84	3189
	TY70	13972
	TY69	619946
	TY68	405040
	TY53	22005
	TY54	19334
	TY52	17690
	TY38	5389
	TY37	27566
	TY36	26858
	TY22	20076
	TY21	18120
	TY20	23981
	TY6	266865
	TY5	34250
	TY4	41679
S. tabernaemontani Water Samples	BG98	66275
	BG99	15104
	BG81	10357
	BG82	13387
	BG83	13166

	BG67	767867
	BG66	1061142
	BG65	150607
	BG51	21082
	BG50	17994
	BG35	1373808
	BG34	22607
	BG33	12511
	BG19	37191
	BG18	956219
	BG17	16717
	BG3	359479
	BG2	834455
	BG1	705933
	BG97	26244
Unplanted Water Samples	CW111	6165
	CW110	11762
	CW109	79614
	CW63	17358
	CW62	11428
	CW47	550928
	CW61	12621
	CW46	803351
	CW45	1159537
	CW31	788779
	CW30	656905
	CW29	718148
	CW15	4697
	CW14	1004238
	CW13	29203
Influent	Inf	24237
Wood chips Biofilm Samples	BioB3	21042
	BioB32	9133
	BioB2	26908
	BioB22	18049
	BioB1	24662
	BioB12	31550
	BioCG3	929157
	BioCG32	17225
	BioCG2	35360
	BioCG22	29673
	BioCG1	50715
	BioCG12	34185
	BioCT3	23116
	BioCT32	18005
	BioCT2	54811
	BioCT22	35546
	BioCT1	43594
	BioCT12	27429
	BioSG3	21775
	BioSG32	21145
	BioSG2	49672
	BioSG22	29626

	BioSG1	42468
	BioSG12	28105
	BioUP3	41321
	BioUP32	26482
	BioUP2	64875
	BioUP22	29065
	BioUP1	82342
	BioUP12	45344
Roots Biofilm Samples	BioSGR2	22804
	BioSGR1	18296
	BioCTR2	20633
	BioCTR1	9776
	BioCGR2	34455
	BioCGR1	18374
	BioBR2	20885
	BioBR1	21566

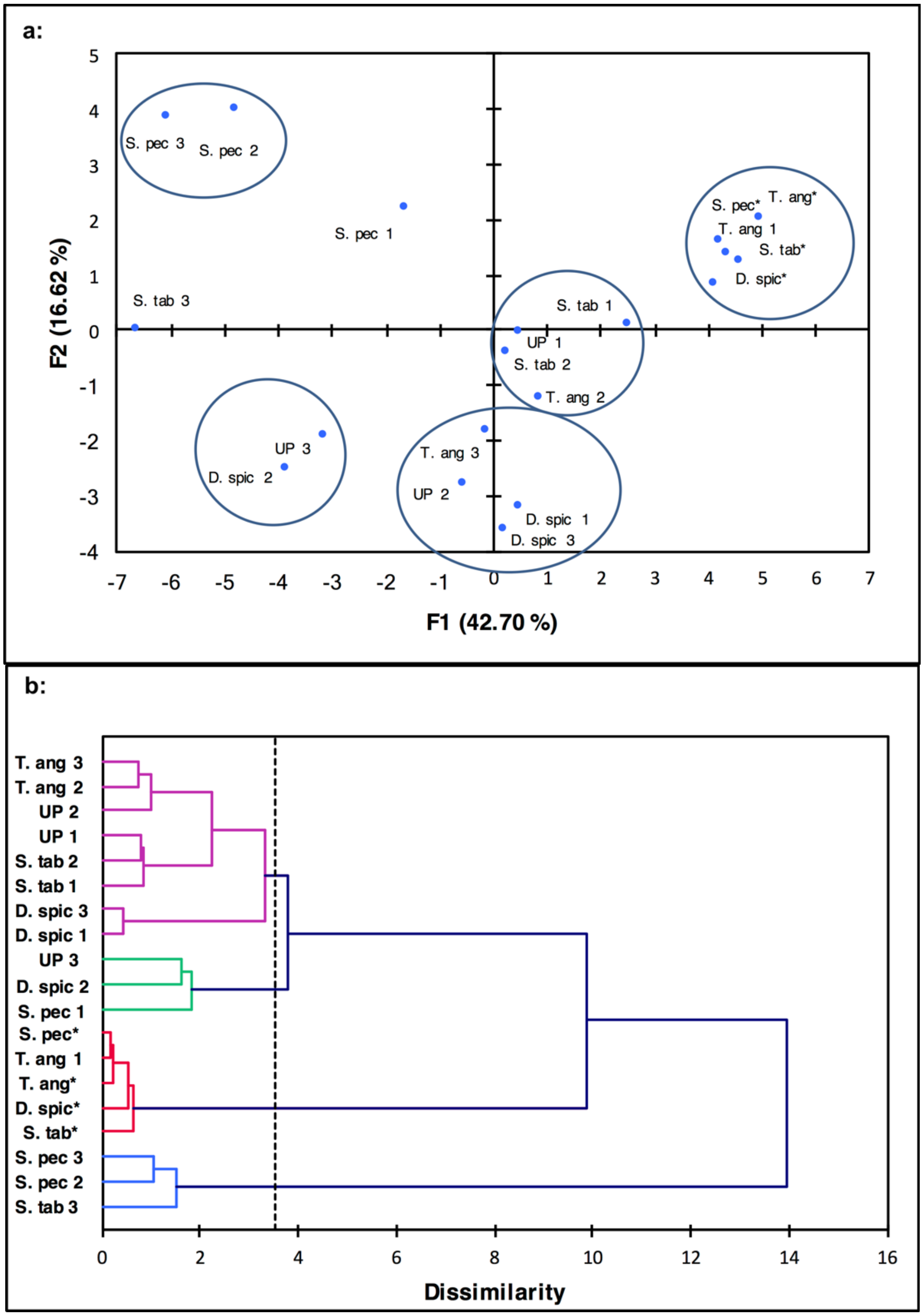

Figure S1. Multivariate analysis for wood-chip and root samples based on the carbon utilization patterns (CSUP). In wood chip samples, 1, 2 and 3 represent top, middle and bottom depths respectively; UP is the unplanted unit and asterisk (*) marks the root samples. **a**: Biofilm samples: Principle component analysis (PCA). The circles show grouped samples based on similarities in their carbon utilization patterns (CSUP); **b**: Biofilm samples: Agglomerative hierarchical clustering (AHC).

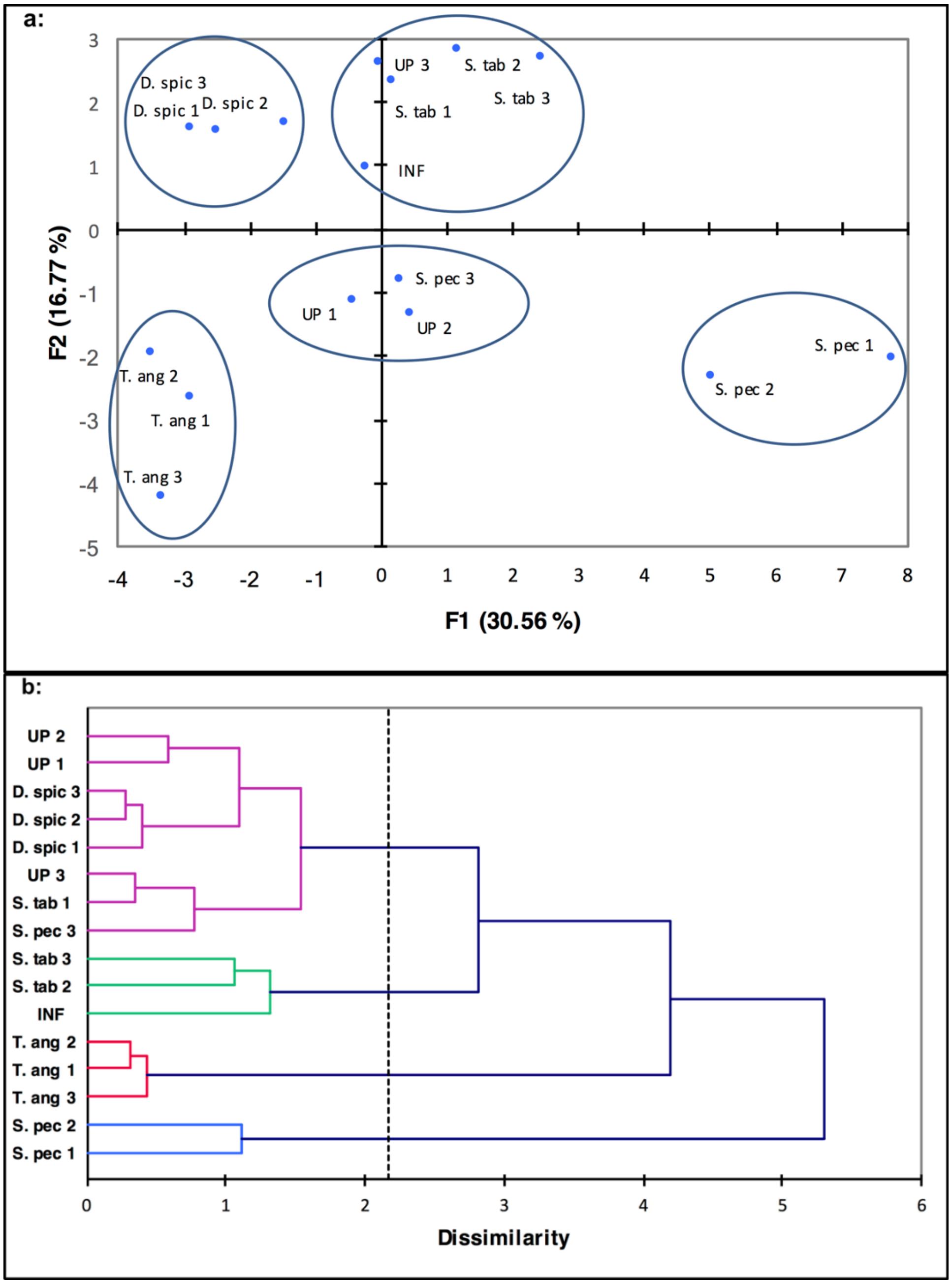

Figure S2. Multivariate analysis for the interstitial water samples based on the carbon utilization patterns (CSUP). 1, 2 and 3 represent top, middle and bottom depths respectively; UP is the unplanted unit and INF is the influent sample; **a**: Interstitial water samples: Principle component analysis (PCA). The circles show grouped samples based on similarities in their carbon utilization patterns (CSUP); **b**: Interstitial water samples: Agglomerative hierarchical clustering (AHC).

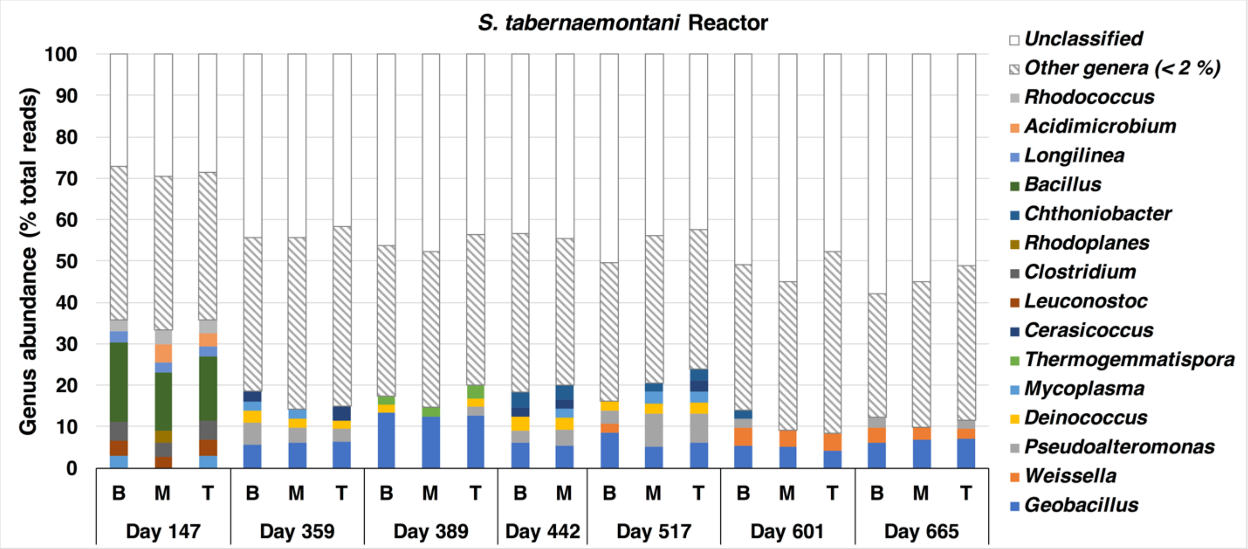

Figure S3. Temporal change of relative bacterial composition at genus level in the *S. tabernaemontani* reactor's interstitial water samples. 'Other genera' is the sum ratios of genera with less than 2% abundance. B, M and T represent bottom (60 cm), middle (40 cm) and top (20 cm) depths respectively.

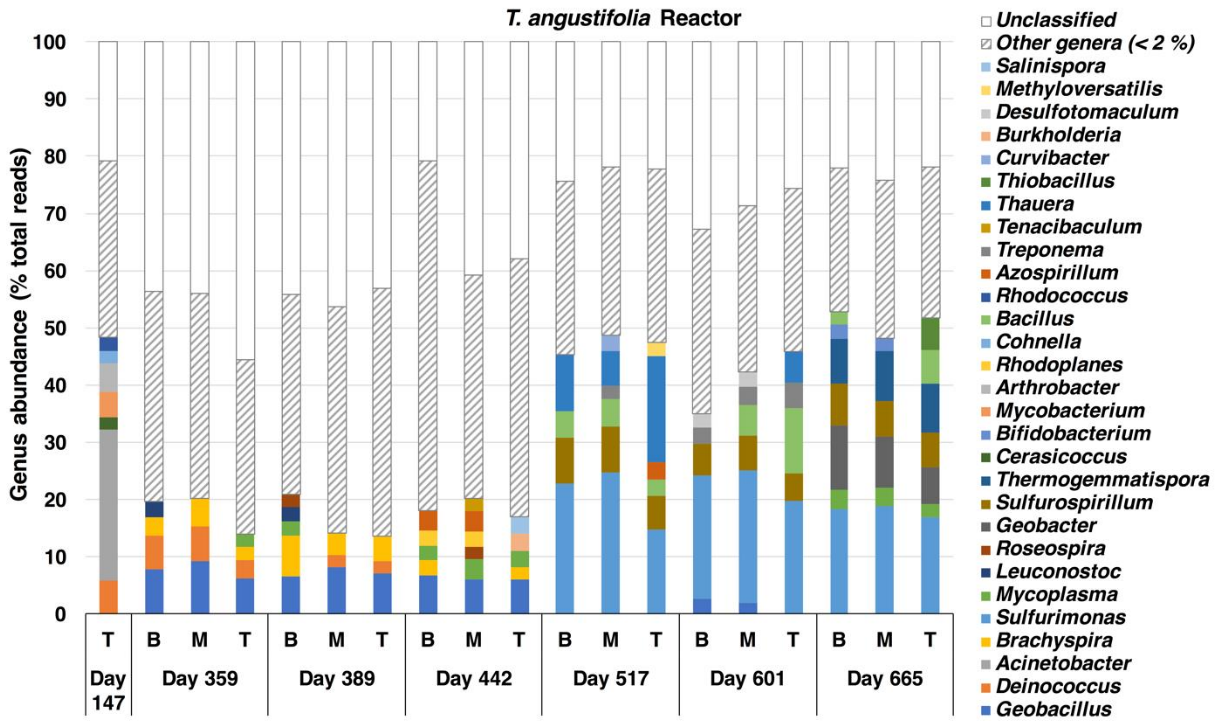

Figure S4. Temporal change of relative bacterial composition at the genus level in the *T. angustifolia* reactor's interstitial water samples. 'Other genera' is the sum ratios of genera with less than 2% abundance. B, M and T represent bottom (60 cm), middle (40 cm) and top (20 cm) depths respectively.

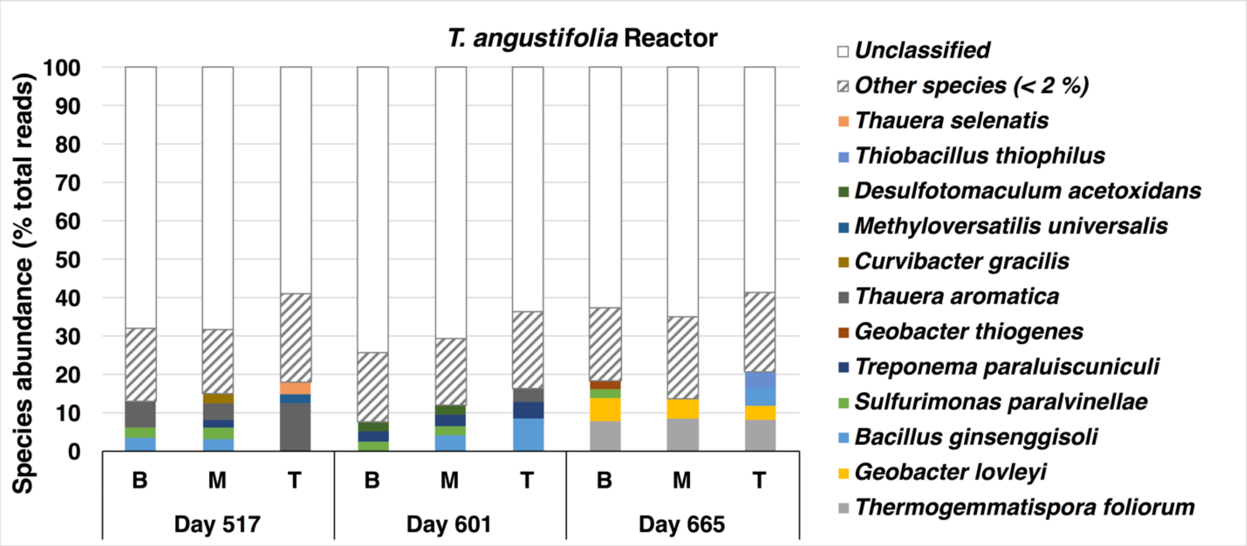

Figure S5. Temporal change of relative bacterial composition at the species level in the *T. angustifolia* reactor's interstitial water samples. 'Other species' is the sum ratios of species with less than 2% abundance. B, M and T represent bottom (60 cm), middle (40 cm) and top (20 cm) depths respectively.

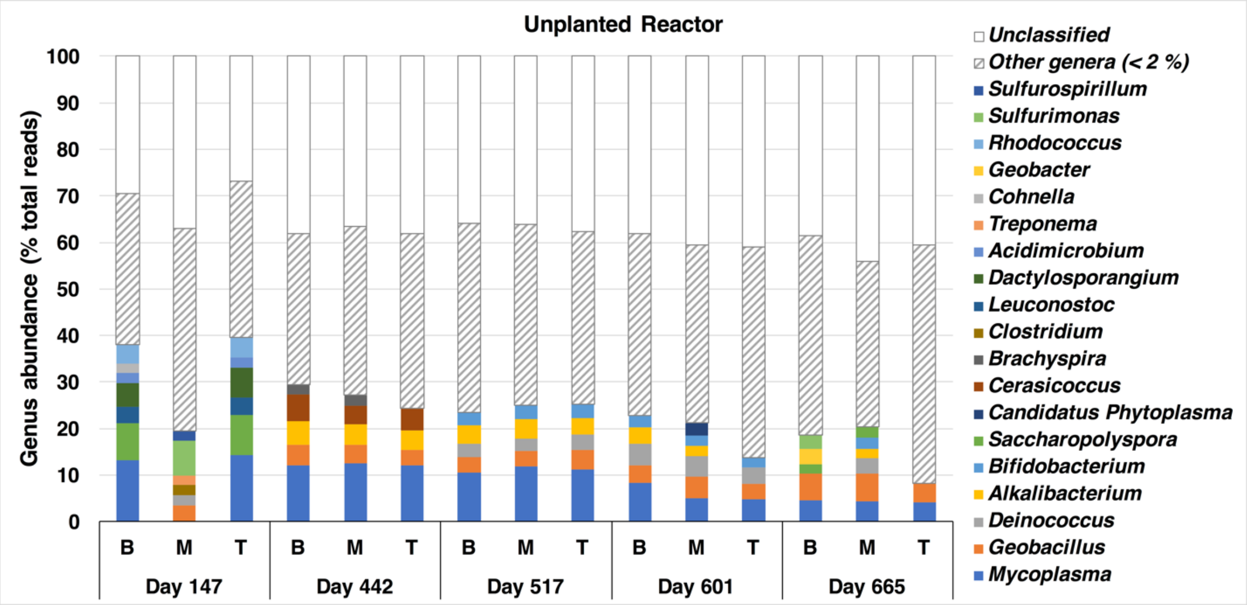

Figure S6. Temporal change of relative bacterial composition at the genus level in the unplanted reactor's interstitial water samples. 'Other genera' is the sum ratios of genera with less than 2% abundance. B, M and T represent bottom (60 cm), middle (40 cm) and top (20 cm) depths respectively.

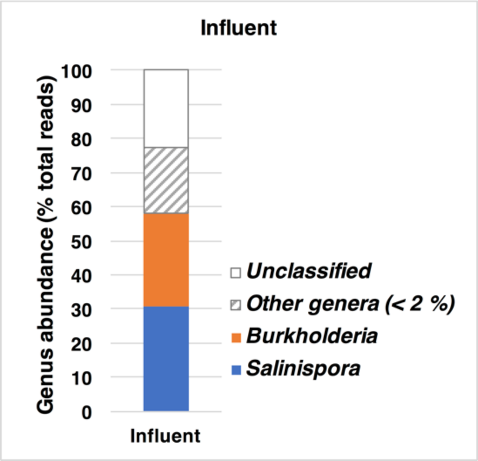

Figure S7. Relative bacterial composition at the genus level in the influent sample collected on Day 442. ’Other genera’ is the sum ratios of genera with less than 2% abundance.

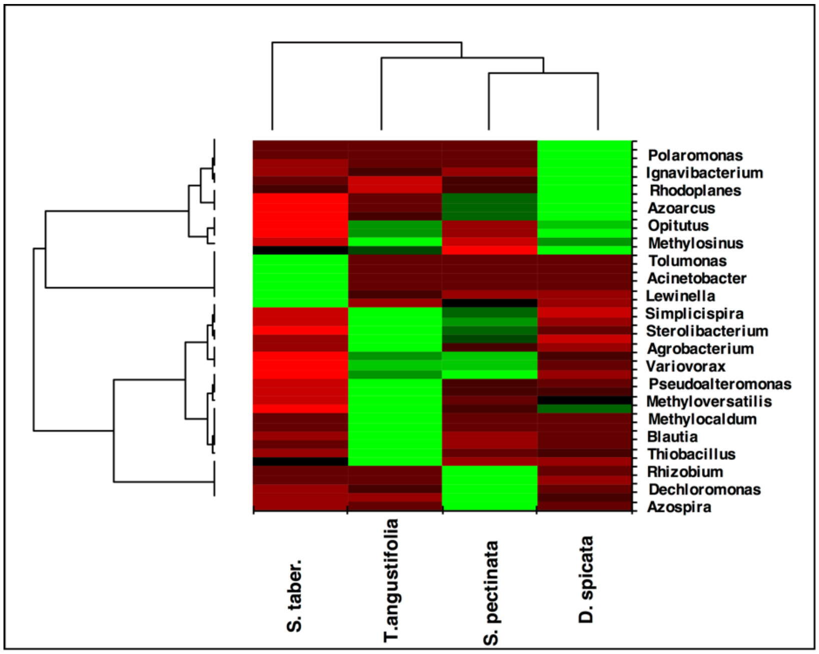

Figure S8. Heatmap and dendrogram of the biofilm from roots, based on the abundance of the microbial community at the genus level. A non-specific filtering for interquartile range <0.25 was used. Only the main genera are shown for clarity and to illustrate the overall similarity between reactors.

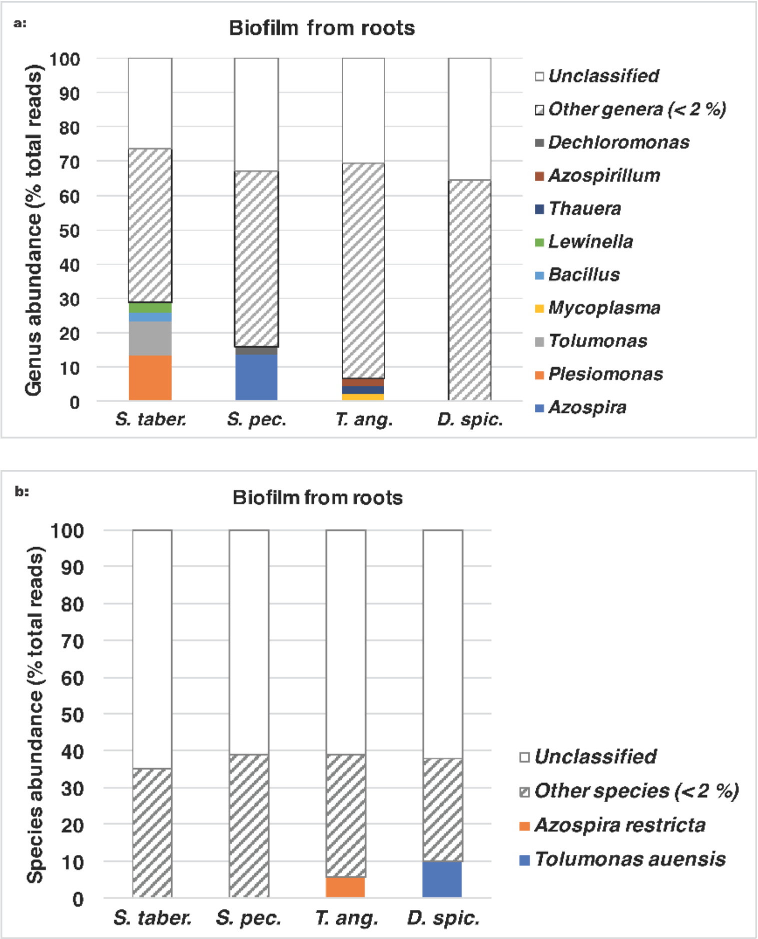

Figure S9. Relative bacterial composition at **a**: the genus level, and **b**: at the species level in the biofilm root samples. 'Other genera' or 'Other species' is the sum ratios of genera or species with less than 2% abundance, respectively.
